# Dynamic changes of complete blood cell count parameters among airport workers during the COVID-19 pandemic in Chongqing, China: A retrospective longitudinal study

**DOI:** 10.1016/j.heliyon.2024.e32734

**Published:** 2024-06-08

**Authors:** Baige Cao, Yingchao Li, Jinfang Xu, Yinan Zhang, Congrong Wang

**Affiliations:** a Department of Endocrinology & Metabolism, Shanghai Fourth People's Hospital, School of Medicine, Tongji University, Shanghai, China; b Department of First Aid Center, Chongqing Jiangbei International Airport, Chongqing, China; c Department of Health Statistics, Second Military Medical University, Shanghai, China; d The Metabolic Disease Biobank, Shanghai Sixth People's Hospital Affiliated to Shanghai Jiao Tong University School of Medicine, Shanghai, China

**Keywords:** COVID-19, Airport staff, Complete blood count, China

## Abstract

**Background and aims:**

This study aimed to examine the dynamic changes in the complete blood counts of airport staff from 2019 to 2021 and assess the impact of the coronavirus disease 2019 (COVID-19) on their overall health status during the first pandemic wave.

**Materials and methods:**

A total of 2144 airport staff members from Chongqing Jiangbei International Airport who underwent health examinations for three consecutive years from 2019 to 2021 were recruited for this study. Venous blood samples were collected for a complete blood cell count.

**Results:**

Changes were observed in blood routine parameters from airport staff over three consecutive years. After adjusting for age, body mass index, and systolic blood pressure, the red blood cell count decreased consecutively during the COVID-19 pandemic. Hemoglobin and basophil counts decreased significantly during COVID-19 year 1. Lymphocyte and platelet counts decreased, whereas the monocyte-to-lymphocyte ratio increased in COVID-19 year 2. However, the white blood cell count, neutrophil count, neutrophil-to-lymphocyte ratio, and eosinophil count did not change from 2019 to 2021.

**Conclusion:**

This study showed changes in complete blood counts in frontline airport workers, especially men, during the COVID-19 pandemic. Therefore, paying more attention to the overall health conditions and immune function of airport staff engaged in intensive work is necessary.

## Abbreviations

COVID-19: Coronavirus disease 2019
BMI: Body mass index
SBP: Systolic blood pressure
RBC: Red blood cells
MLR: Monocyte-to-lymphocyte ratio
WBC: White blood cells
NLR: Neutrophil-to-lymphocyte ratio
PLT: Platelet
CI: Confidence interval
IQR: Interquartile range.

## 1 Introduction

The coronavirus disease 2019 (COVID-19) outbreak created an unprecedented threat to public health. The crisis drastically affected the world, particularly the airline sector. Air transportation provides a worldwide network that is a strong pillar for developing global business and tourism. From 2020 to 2021, the Chongqing Jiangbei International Airport had approximately 35 million passengers annually, ranking fourth among all airports in mainland China regarding passenger throughput over two consecutive years. In public places with a large population flow, airport workers were inevitably under tremendous pressure to prevent the spread of COVID-19 within or beyond China. In 2020, the World Health Organization proposed that airports should have contingency plans and arrangements for managing the COVID-19 pandemic, such as measuring the body temperature of passengers and conducting a questionnaire for the early detection of symptoms [1], which would increase person-to-person contact. Previous studies reported that the risk of infection among airport staff was equivalent to that among healthcare workers [2]. Owing to higher occupational risk exposure, international airport staff were defined as a key population [3]. The Civil Aviation Administration of China also proposed stricter epidemic control measures for airport workers, such as receiving a COVID-19 vaccine booster, daily nucleic acid testing, and closed-loop management. Moreover, the pandemic-related coping strategies changed lifestyle habits such as diet, physical activity, and sleep behaviors, which might have led to metabolic dysfunction, depression, stress, and anxiety [4,5]. Therefore, the physical and emotional health of airport staff was significantly challenged during the COVID-19 pandemic.

A complete blood count is an essential blood test to evaluate an individual's overall health conditions [6]. Red blood cells (RBC) are produced in the bone marrow and contain hemoglobin, which is important for evaluating the oxygen transport capacity of blood. White blood cells (WBC) can be used as biomarkers for inflammation, immune response, and other hematological malignancies. Platelets (PLT) are also a major blood constituent and play a key role in coagulation, thrombosis, and inflammation [7]. It is well established that the immune system is sensitive to psychological stress [8]. Long-term stress has been shown to induce the reduction of lymphocyte count [9,10]. Instead, Yang et al. found that lymphocyte counts increased, whereas the neutrophil-to-lymphocyte ratio (NLR) and monocyte-to-lymphocyte ratio (MLR) decreased in the peripheral blood of frontline healthcare workers 10 days after they went to support Wuhan during the first epidemic wave of COVID-19 in China [11]. However, the impact of COVID-19 on airport workers' health remains largely unknown.

Here, we aimed to study the dynamic changes in the complete blood count of airport staff between 2019 and 2021 and assess the relationship between the COVID-19 pandemic and their overall health status.

## 2 Materials and Methods

### 2.1 Study design and participants

**Fig. 1:**
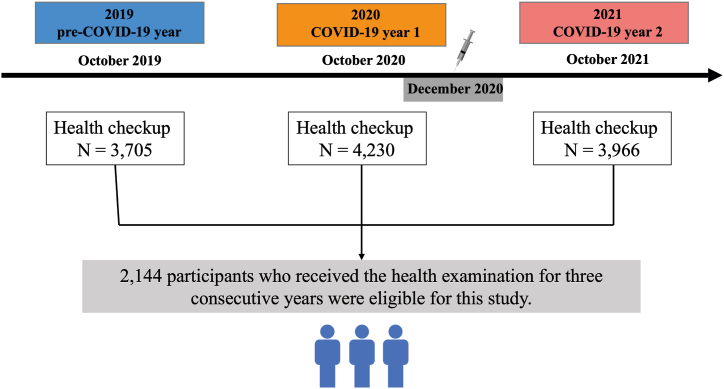
Study flowchart.

This retrospective longitudinal study included airport ground staff and public security officers from Chongqing Jiangbei International Airport who underwent checkups at the First Aid Center of Chongqing Jiangbei International Airport between October 2019 and October 2021. Repeat visitors who underwent health checkups before the COVID-19 pandemic (October 2019, defined as pre-COVID-19), during the first year (October 2020, defined as COVID-19 year 1), and second year of the pandemic (October 2021, defined as COVID-19 year 2) were included in this study. All participants had SARS-CoV-2 negative results from daily nucleic acid tests during the study period. Participants with severe infections, malignant diseases, acute metabolic complication, and prior medical history with severe mental illnesses were excluded from the study. A total of 2144 Participants (1360 men and 783 women) who underwent health examinations for three consecutive years were enrolled in this study (Fig. 1).

### 2.2 Health checkup variables

Demographic information, including age, sex (man or woman), type of work (night-shift or non-shift work), vaccination history (yes or no), and type of COVID-19 vaccine (Sinopharm, Sinovac, or ZF2001), was collected by a trained nurse. All participants were administered the first shots of the COVID-19 vaccine in December 2020, and 99.5% received a second booster dose before May 2021. Night-shift work was defined as a minimum of two night shifts per week over three years. The participants underwent a physical examination by trained nurses. Height and weight were measured in light clothing without shoes. Body mass index (BMI) was calculated as weight divided by height squared (kg/m^2^). Overweight was defined as a BMI of 24–28 kg/m2, and obese was defined as a BMI >28 kg/m^2^ [12]. Blood pressure was measured in a seated position after 10 min of rest. Venous blood samples were collected from participants after overnight fasting. Complete blood cell counts were performed using the Mindray BC-5180 Auto Hematology Analyzer (Mindray Bio-Medical Electronics Co., Ltd., Shenzhen, China). Blood test data included WBC count, neutrophil count, monocyte count, lymphocyte count, NLR, MLR, eosinophil count, basophil count, RBC count, hemoglobin level, and PLT count. The NLR was calculated as the neutrophil count divided by the lymphocyte count. Similarly, MLR was calculated as the monocyte count divided by the lymphocyte count.

### 2.3 Statistical analysis

Data are presented as mean ± standard deviation for continuous variables, median (interquartile range) (IQR) for non-normally distributed variables, and proportions for categorical variables. Comparisons of continuous variables among the different groups were performed using repeated-measures ANOVA. Chi-square tests were used to compare categorical data across groups, and the Cochran–Mantel–Haenszel test was used for ordinal categorical variables. Each laboratory test indicator was classified into three ordinal categories according to the reference range. A multiple linear regression model was used to analyze the annual laboratory test indicator results after adjusting for age, BMI, and systolic blood pressure (SBP). Furthermore, subgroup analyses stratified by age, sex, and type of work were performed using multiple linear regression after adjusting for the same factors. A P value of <0.05 (two-tailed) was considered statistically significant. Analyses were performed using the SAS System for Windows, version 9.1 (SAS Institute, Inc., Cary, NC, USA).

## 3 Results

The demographic characteristics of the 2144 participants are presented in Table 1. The median age of participants at baseline was 34.6 years. Among the 2144 participants, 1360 (63.43%) were men, and 784 (36.57%) were women. The BMI and blood pressure increased significantly from 2019 to 2021.

**Table 1:** Demographics characteristics of the participants (N = 2144).

	Year			
	2019	2020	2021	*P* value
	(pre-COVID-19 year)	(COVID-19 year 1)	(COVID-19 year 2)	
Age, years	34.6 (29.3–43.1)	35.6 (30.4–44.1)	36.6 (31.4–45.1)	<0.0001
Age, years, n (%)				<0.0001
<40	1490 (69.50)	1423 (66.37)	1353 (63.11)	
≥40	654 (30.50)	721 (33.63)	791 (36.89)	
Sex				
Man	1360 (63.43)	1360 (63.43)	1360 (63.43)	
Woman	784 (36.57)	784 (36.57)	784 (36.57)	
Body mass index, kg/m^2^	23.0 (20.8–25.3)	23.1 (21.0–25.4)	23.4 (21.2–25.6)	0.0067
Body mass index, kg/m^2^, n (%)				0.0716
<24	1312 (61.19)	1265 (59.00)	1220 (56.90)	
24–28	674 (31.44)	720 (33.58)	746 (34.79)	
>28	158 (7.37)	159 (7.42)	178 (8.30)	
Systolic blood pressure, mmHg	120.0 (110.0–131.0)	121.0 (110.0–131.0)	122.0 (112.0–132.0)	0.0038
Diastolic blood pressure, mmHg	75.0 (68.0–83.0)	75.0 (69.0–83.0)	76.0 (68.0–84.0)	0.0090

Data are median (interquartile range) or n (%).

**Fig. 2:**
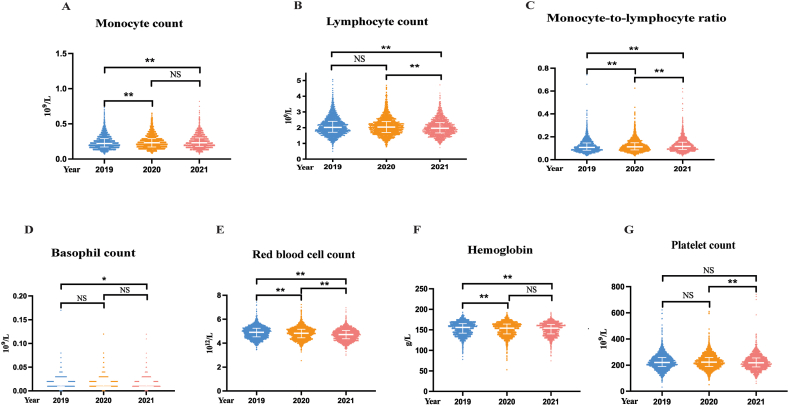
Dynamic changes of complete blood count parameters from 2019 to 2021. A. Monocyte count; B. Lymphocyte count; C. monocyte-to-lymphocyte; D. Basophil count; E. Red blood cell count; F. Hemoglobin; G. Platelet count. *P < 0.05; **P < 0.01; NS: No significance.

Table 2 and Fig. 2 (A-G) show the dynamic changes in blood routine parameters from airport staff over three years. A significant consecutive increase in the MLR and a decrease in the RBC count were observed (Table 2, Fig. 2 C, E). After adjusting for age, BMI, and SBP, the association between RBC count and COVID-19 pandemic remained significant. For MLR, a positive correlation was observed only from 2020 to 2021 and from 2019 to 2021 after adjustment (Table 3). Notably, monocyte count was significantly increased [median (IQR): 0.23 × 10^9^/L (0.17–0.29 × 10^9^/L) vs. 0.22 × 10^9^/L (0.17–0.29 × 10^9^/L), P = 0.0067] while hemoglobin was decreased [median (IQR): 154.00 g/dL (140.00–164.00 g/dL) vs. 156.00 g/dL (142.00–165.00 g/dL), P = 0.0019] in COVID-19 year 1 compared with pre-COVID-19 year, but they were unchanged during the COVID-19 pandemic (Table 2, Fig. 2 A, F). This finding was consistent when we assessed hemoglobin levels using the adjusted model in a linear regression analysis. However, the positive correlation between monocyte count and COVID-19 diminished after adjustment (all P > 0.05, Table 3). The lymphocyte and PLT counts were comparable between 2019 and 2020 but decreased significantly during the COVID-19 pandemic (Fig. 2 B, G), and these associations remained significant even after adjusting for the same factors (Table 3). The basophil count dropped in COVID-19 year 2 compared with the pre-COVID-19 year (Fig. 2 D). A negative association was also found between 2019 and 2020 after controlling for the abovementioned confounding factors (Table 3). However, WBC count, neutrophil count, NLR, and eosinophil count did not change from 2019 to 2021.

**Table 2:** Complete blood count parameters of the participants from 2019 to 2021 (N = 2144).

	Year			
	2019	2020	2021	*P* value
	(pre-COVID-19 year)	(COVID-19 year 1)	(COVID-19 year 2)	
White blood cell count, × 10^9^/L	6.2 (5.3–7.2)	6.2 (5.3–7.2)	6.1 (5.2–7.1)	0.1324
White blood cell count, × 10^9^/L, n (%)				0.2497
<4	58 (2.71)	41 (1.91)	61 (2.85)	
4–10	2035 (94.92)	2057 (95.94)	2028 (94.59)	
>10	51 (2.38)	46 (2.15)	55 (2.57)	
Neutrophil count, × 10^9^/L	3.6 (3.0–4.5)	3.7 (3.0–4.5)	3.7 (3.0–4.5)	0.3689
Neutrophil count, × 10^9^/L, n (%)				0.4323
<4	44 (2.05)	35 (1.63)	43 (2.01)	
4–10	2056 (95.90)	2075 (96.78)	2053 (95.76)	
>10	44 (2.05)	34 (1.59)	48 (2.24)	
Monocyte count, × 10^9^/L	0.22 (0.17–0.29)	0.23 (0.17–0.29)	0.23 (0.18–0.30)	0.0252
Monocyte count, × 10^9^/L, n (%)				0.0972
<0.12	73 (3.40)	47 (2.19)	59 (2.75)	
0.12–1.2	2070 (96.55)	2097 (97.81)	2085 (97.25)	
>1.2	1 (0.05)	0 (0.00)	0 (0.00)	
Lymphocyte count, × 10^9^/L	2.02 (1.71–2.41)	2.04 (1.70–2.39)	1.97 (1.67–2.32)	<0.0001
Lymphocyte count, × 10^9^/L, n (%)				0.0555
<0.8	4 (0.19)	1 (0.05)	1 (0.05)	
0.8–4	2129 (99.30)	2128 (99.25)	2139 (99.77)	
>4	11 (0.51)	15 (0.70)	4 (0.19)	
Neutrophil-to-lymphocyte ratio	1.81 (1.43–2.28)	1.81 (1.46–2.28)	1.84 (1.49–2.30)	0.0659
Monocyte-to-lymphocyte ratio	0.11 (0.08–0.15)	0.11 (0.09–0.15)	0.12 (0.09–0.15)	<0.0001
Eosinophil count, × 10^9^/L	0.11 (0.07–0.19)	0.11 (0.07–0.19)	0.12 (0.07–0.19)	0.3097
Eosinophil count, × 10^9^/L, n (%)				0.8741
＜0.02	8 (0.37)	9 (0.42)	6 (0.28)	
0.02–0.5	2092 (97.57)	2097 (97.81)	2100 (97.95)	
＞0.5	44 (2.05)	38 (1.77)	38 (1.77)	
Basophil count, × 10^9^/L	0.02 (0.01–0.02)	0.02 (0.01–0.02)	0.02 (0.01–0.02)	0.0054
Basophil count, × 10^9^/L, n (%)				0.7787
＜0	0 (0.00)	0 (0.00)	0 (0.00)	
0–0.1	2143 (99.95)	2143 (99.95)	2142 (99.91)	
＞0.1	1 (0.05)	1 (0.05)	2 (0.09)	
Red blood cell count, × 10^12^/L	4.91 (4.53–5.24)	4.83 (4.45–5.16)	4.72 (4.36–5.05)	<0.0001
Red blood cell count, × 10^12^/L, n (%)				<0.0001
<3.5	3 (0.14)	9 (0.42)	8 (0.37)	
3.5–5.5	1912 (89.18)	1949 (90.90)	2038 (95.06)	
>5.5	229 (10.68)	186 (8.68)	98 (4.57)	
Hemoglobin, g/dL	156.00 (142.00–165.00)	154.00 (140.00–164.00)	154.00 (140.00–163.00)	<0.0001
Hemoglobin, g/dL, n (%)				0.0049
<110	26 (1.21)	28 (1.31)	28 (1.31)	
110–160	1238 (57.74)	1318 (61.47)	1356 (63.25)	
>160	880 (41.04)	798 (37.22)	760 (35.45)	
Platelet count, × 10^9^/L	221.00 (190.00–258.00)	223.00 (190.00–259.00)	218.00 (186.00–255.00)	<0.0001
Platelet count, × 10^9^/L, n (%)				0.7951
<100	10 (0.47)	9 (0.42)	7 (0.33)	
100–300	1958 (91.32)	1949 (90.90)	1970 (91.88)	
>300	176 (8.21)	186 (8.68)	167 (7.79)	

Data are median (interquartile range) or n (%).

**Table 3:** Associations of changes in complete blood count parameters and study periods.

	2019 vs. 2020		2020 vs. 2021		2019 vs. 2021	
	β (95%CI)	*P* value	β (95%CI)	*P* value	β (95%CI)	*P* value
Monocyte count, × 10^9^/L	0.00 (−0.01, 0.01)	0.4671	0.00 (−0.00, 0.01)	0.4261	0.00 (−0.00, 0.01)	0.2294
Lymphocyte count, × 10^9^/L	−0.00 (−0.03, 0.03)	0.9596	−0.06 (−0.09, −0.03)	0.0002	−0.03 (−0.05, −0.01)	0.0003
Monocyte-to-lymphocyte ratio	0.00 (−0.00, 0.01)	0.1578	0.00 (0.00, 0.01)	0.0065	0.00 (0.00, 0.00)	<0.0001
Basophil count, × 10^9^/L	−0.01 (−0.02, −0.00)	0.0228	−0.00 (−0.01, 0.01)	0.8241	−0.01 (−0.01, −0.00)	0.0112
Red blood cell count, × 10^12^/L	−0.07 (−0.10, −0.05)	<0.0001	−0.13 (−0.15, −0.10)	<0.0001	−0.10 (−0.11, −0.09)	<0.0001
Hemoglobin, g/dL	−1.79 (−2.64, −0.94)	<0.0001	−0.76 (−1.61, 0.09)	0.0807	−1.27 (−1.69, −0.85)	<0.0001
Platelet count, × 10^9^/L	3.19 (−0.14, 6.51)	0.0603	−3.68 (−6.98, −0.38)	0.0290	−0.25 (−1.94, 1.45)	0.7756

Adjusted for age, body mass index and systolic blood pressure. β, beta coefficient. 95% CI, 95% confidence interval.

**Fig. 3:**
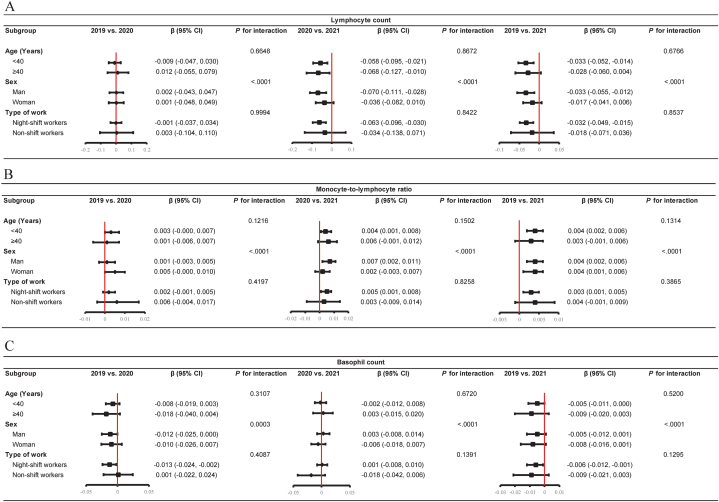
Adjusted subgroup analysis for lymphocyte count, monocyte-to-lymphocyte ratio, basophil count from 2019 to 2021. A. Lymphocyte count; B. Monocyte-to-lymphocyte ratio; C. Basophil count; All the results were adjusted for age, body mass index, and systolic blood pressure. β, beta coefficient. 95% CI, 95% confidence interval.

**Fig. 4:**
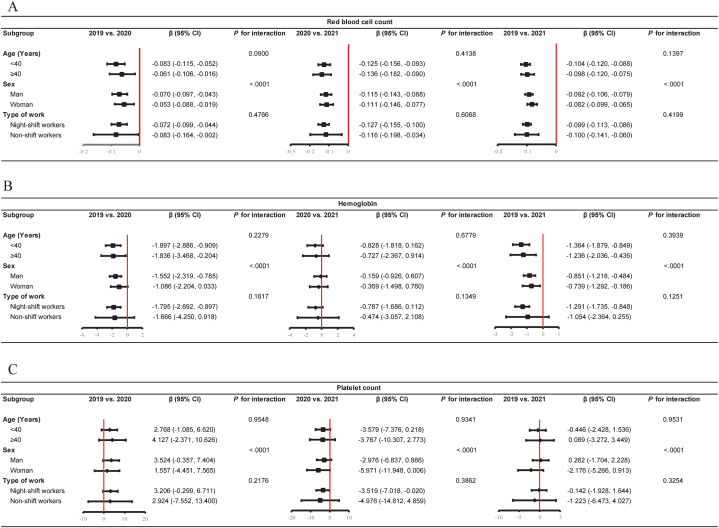
Adjusted subgroup analysis for red blood cell count, hemoglobin; platelet count from 2019 to 2021. A. Red blood cell count; B. Hemoglobin; C. Platelet count. All the results were adjusted for age, body mass index, and systolic blood pressure. β, beta coefficient. 95% CI, 95% confidence interval.

Subgroup analysis was performed to explore the robustness of the association between lymphocyte count, MLR, basophil count, RBC count, hemoglobin level, PLT count, and the COVID-19 pandemic stratified by age, sex, and type of work (Fig. 3 A-C, Fig. 4 A-C). Interaction tests showed that these associations were dependent on sex (Ps for interaction <0.01). Interestingly, the RBC count significantly differed in men and women over the three consecutive years (Fig. 4 A). In addition, the effect of the COVID-19 pandemic on MLR (men: β = 0.01, 95% confidence interval [CI], 0.00 to 0.01, P for interaction <0.0001; β = 0.00, 95% CI, 0.00 to 0.01, P for interaction <0.0001), lymphocyte count (men: β = −0.07, 95% CI, −0.11 to −0.03, P for interaction <0.0001; β = −0.03, 95% CI, −0.06 to −0.01, P for interaction <0.0001) was more significant on men than women from 2020 to 2021 and 2019–2021 (Fig. 3 A, B). In addition, the negative association between COVID-19 and hemoglobin was more significant in men (men: β = −1.55, 95% CI -2.32 to −0.79, P for interaction <0.0001; β = −0.85, 95% CI -1.22 to −0.48, P for interaction <0.0001) from 2019 to 2020 and 2019–2021 (Fig. 4 B). However, all associations between lymphocyte count, MLR, basophil count, RBC count, hemoglobin level, PLT count, and the COVID-19 pandemic were consistent in the age and type of work subgroups (P for interaction >0.05 for all covariates).

## 4 Discussion

To the best of our knowledge, this is the first observational study on the relationship between the COVID-19 pandemic and blood routine parameters in airport workers. Linear regression analysis showed a significant consecutive decrease in RBC count during the COVID-19 pandemic. Hemoglobin and basophil counts decreased significantly during COVID-19 year 1. Lymphocyte and platelet counts decreased, whereas MLR increased in COVID-19 year 2. Moreover, subgroup analyses showed stronger associations between the pandemic and blood routine parameters in men, especially hemoglobin, lymphocyte, and MLR levels.

The COVID-19 pandemic posed significant challenges to the daily work life of frontline airport workers [13], including the requirements for daily nucleic acid testing and closed-loop management. In addition, they were required to measure the temperature of passengers and conduct epidemiological surveys for early detection of symptoms. Decreases in RBC and hemoglobin levels were observed among airport workers. Previous studies have reported that excessive physical exercise induces the physical destruction of RBCs, also causing a decrease in hemoglobin [14], mainly caused by plasma volume expansion [15]. Moreover, reduced iron absorption in the small intestine and decreased iron export to the circulation from parenchymal cells and macrophages may be another reason for these reduced levels [16]. It was found that individuals who underwent eight weeks of mat Pilates training had higher plasma volume variations and lower hemoglobin values than the control group [17]. However, whether the decrease in RBC and hemoglobin levels in frontline airport workers is driven by excessive physical exercise requires further study.

COVID-19 placed great stress on frontline healthcare workers and caused them to endure psychological burden [18]. Frontline airport workers were in contact with thousands of travelers, thus equating them with the risks faced by workers in the healthcare sector. In this study, some immune parameters of frontline airport workers changed significantly after the COVID-19 pandemic. Lymphocyte count decreased significantly in COVID-19 year 2. A previous study showed that psychological stress could influence the redistribution of leukocytes via catecholaminergic neurotransmitters and corticosterone [19]. An in vivo study showed that stress could induce peripheral lymphocytes to migrate to the bone marrow, which is controlled by the hypothalamic-pituitary-adrenal axis [20]. Moreover, chronic stress decreases the lymphocyte proliferative response [21] and induces lymphocyte apoptosis [22]. Studies on mice have shown that chronic stress induces autophagy, ultimately suppressing the immune system [23].

In addition, the basophil count decreased significantly in COVID-19 year 1 in our study, which is consistent with a study of frontline healthcare workers who assisted patients with COVID-19 [24]. Basophils represent less than 1% of peripheral blood leukocytes but play a critical role in developing immunoglobulin E-mediated chronic allergic inflammation independent of T cells and mast cells [25,26]. Interestingly, Qureshi et al. reported that the stressful state caused by medical student examinations could decrease basophil count [27]. Our unpublished data also showed that frontline airport workers experienced psychological stress similar to frontline healthcare workers. Psychological research can further explain immune system changes in airport workers during the COVID-19 pandemic.

In our study, the PLT count marginally increased in COVID-19 year 1 but dropped significantly in COVID-19 year 2; however, the latter was comparable to pre- COVID-19 year. Previous studies showed that PLT counts can be elevated by strenuous exercise and psychological stress [28,29]. In vitro and in vivo studies showed that epinephrine and norepinephrine activated by psychological stress stimulate PLT activation [30]. However, our results indicated that PLT count was not a significant marker for stress in airport staff (in COVID-19 year 1) but might have been affected by the vaccine (in COVID-19 year 2). This aligns with previous research showing that the PLT count decreased but was within the normal range after receiving the ChAdOx1 nCoV-19 vaccination [31].

Immune responses in men are lower than in women concerning viral infections in animals and humans [32,33]. Moreover, aging is more strongly associated with a higher risk of death in men >30 years, making older men the most vulnerable group [34].

Interestingly, the changes in blood routine parameters in men were more significantly associated with the COVID-19 pandemic in the present study, making them more vulnerable than women. Indeed, reference values for hemoglobin and RBC count showed significant sex differences in the Southwest Chinese population, and these major RBC parameters decreased with age in men [35]. The potential mechanism underlying this sexual dimorphism is complicated; further research is required to explain this variation. Nevertheless, our data suggested that more attention should be paid to male frontline airport workers.

The main advantage of this study was that we investigated the relationship between the COVID-19 pandemic and blood routine parameters in airport workers, thereby providing valuable insights into health monitoring in this high-risk, vulnerable population. In clinical practice, a complete blood count is a convenient and low-cost routine examination that can effectively monitor an individual's overall health and immune status. However, this study has some limitations. First, the lack of a prospective design made it challenging to confirm the causal relationships among the variables. Second, the single-center nature of the study made it difficult to extrapolate our findings to all airport staff. Third, the effects of metabolic diseases, malignant tumors, allergies, and infectious diseases on routine blood parameters could not be ruled out. Nevertheless, participants with severe infections, malignant diseases, acute metabolic complication, and severe mental illnesses, as reported in their medical histories, were not included in this study.

In conclusion, our study showed that changes in complete blood count were found in frontline airport workers, especially men, during the COVID-19 pandemic. Therefore, paying more attention to the overall health conditions and immune function of airport staff engaged in intensive work during the pandemic is necessary.

## Declarations

### 4.1 Ethics statement

This study was approved by the Ethics Committee of Tongji University in 4, 7, 2022 (No. 2022tjdxsy035). Written informed consent was waived due to retrospective anonymized data collection. Participants were also provided a web-based option to opt out if they refused to participate.

## Funding statement

This work was supported by the Shanghai Science and 10.13039/100006180Technology Development Funds, China [Grant Nos. 20ZR1446000, 22410713200], Research Fund from Shanghai Fourth People's Hospital [sykyqd01801, SY-XKZT-2021-1001], Shanghai Research Center for Endocrine and Metabolic Diseases [Grant No. 2022ZZ01002], and the Open Research Project of Shanghai Key Laboratory of Diabetes Mellitus [SHKLD-KF-2101].

## Data availability statement

Data will be made available on request.

## CRediT authorship contribution statement

**Baige Cao:** Writing – review & editing, Writing – original draft, Investigation, Formal analysis. **Yingchao Li:** Writing – review & editing, Writing – original draft, Investigation, Formal analysis. **Jinfang Xu:** Formal analysis. **Yinan Zhang:** Writing – review & editing, Writing – original draft, Supervision, Funding acquisition, Conceptualization. **Congrong Wang:** Writing – original draft, Supervision, Funding acquisition, Conceptualization.

## Declaration of competing interest

The authors declare that they have no known competing financial interests or personal relationships that could have appeared to influence the work reported in this paper.
